# Stimulation by exosomes from hypoxia-preconditioned hair follicle mesenchymal stem cells facilitates mitophagy by inhibiting the PI3K/AKT/mTOR signaling pathway to alleviate ulcerative colitis

**DOI:** 10.7150/thno.96038

**Published:** 2024-07-08

**Authors:** Ning Li, Lei Zhao, Xinyu Geng, Jingyang Liu, Xu Zhang, Ying Hu, Jihan Qi, Hongliang Chen, Jiawei Qiu, Xiaoyu Zhang, Shizhu Jin

**Affiliations:** 1 Department of Gastroenterology and Hepatology, The Second Affiliated Hospital of Harbin Medical University, Harbin 150086, China.; 2 Department of Infectious Diseases, The Second Affiliated Hospital of Harbin Medical University, Harbin 150086, China.

**Keywords:** Hair follicle mesenchymal stem cell, Exosome, PI3K/AKT/mTOR signaling pathway, Mitophagy, Ulcerative colitis

## Abstract

**Background:** Ulcerative colitis (UC) is an intestinal inflammatory disease that is strongly associated with mitochondrial damage and dysfunction as well as mitophagy and lacks of satisfactory treatments. Hair follicle mesenchymal stem cell (HF-MSC)-derived exosomes owe benefit effectiveness on inflammatory therapies. Hypoxia-preconditioned HF-MSCs exhibit enhanced proliferation and migration abilities, and their exosomes exert stronger effects than normal exosomes. However, the therapeutic function of Hy-Exos in UC is unknown.

**Methods:** The inflammation model was established with LPS-treated MODE-K cells, and the mouse UC model was established by dextran sulfate sodium (DSS) administration. The therapeutic effects of HF-MSC-derived exosomes (Exos) and hypoxia-preconditioned HF-MSC-derived exosomes (Hy-Exos) were compared *in vitro* and *in vivo*. Immunofluorescence staining and western blotting were used to explore the effects of Hy-Exos on mitochondrial function, mitochondrial fission and fusion and mitophagy. MiRNA sequencing analysis was applied to investigate the differences in components between Exos and Hy-Exos.

**Results:** Hy-Exos had a better therapeutic effect on LPS-treated MODE-K cells and DSS-induced UC mice. Hy-Exos promoted colonic tight junction proteins expression, suppressed the oxidative stress response, and reduced UC-related inflammatory injury. Hy-Exos may exert these effects via miR-214-3p-mediated inhibition of the PI3K/AKT/mTOR signaling pathway, maintenance of mitochondrial dynamic stability, alleviation of mitochondrial dysfunction and enhancement of mitophagy.

**Conclusion:** This study revealed a vital role for Hy-Exos in suppressing inflammatory progression in UC and suggested that miR-214-3p is a potential critical target for Hy-Exos in alleviating UC.

## Introduction

The pathogenesis of ulcerative colitis (UC), which is a nonspecific inflammatory bowel disease, is associated with a variety of factors, mainly including genetic susceptibility, intestinal flora imbalance, intestinal epithelial barrier injury, immune dysregulation and environmental factors 1, 2. When the intestinal epithelial barrier is compromised, its ability to sense and remove harmful bacteria is reduced, which ultimately exacerbates inflammation of the intestinal mucosa 3. The incidence of UC has increased annually in recent years, and the proportion of young patients has also increased 4. 5-Aminosalicylic acid, hormones, immunosuppressants, and biologics are the most common nonsurgical treatments for UC. The long-term application of these drugs is accompanied by many adverse effects, additionally, some patients who receive biologic therapies do not respond to these drugs, and the therapeutic effects decrease over time in some patients 5. Surgical treatment is the ultimate solution for curing UC, but complications, such as postoperative stricture and anastomotic fistula, seriously impact on patients' quality of life 6. Therefore, it is crucial to explore the treatment of UC.

Mesenchymal stem cells (MSCs), which have the potential for self-renewal and multidirectional differentiation, have good therapeutic effects on a variety of diseases. Hair follicle mesenchymal stem cells (HF-MSCs) are abundant, easily accessible with little harm to donors, and have strong proliferative abilities and good application prospects 7, 8. MSCs exert therapeutic effects mainly via paracrine mechanisms 9. Exosomes are 40-160 nm extracellular vesicles that carry a variety of proteins, mRNAs, miRNAs and lipids that mediate the paracrine effects of MSCs 10, 11. Exosomes are important tools for intercellular communication, and they will likely become a novel “cell-free” therapy. Studies have demonstrated that MSC-derived exosomes can promote the repair of damaged intestinal tissue in UC. Moreover, hypoxia-preconditioned MSCs exhibit enhanced proliferative and migratory abilities as well as improved antioxidant properties, and their exosomes exert stronger effects than normal exosomes 12, 13.

The PI3K/AKT/mTOR signaling pathway is essential for UC pathogenesis. PI3K regulates energy metabolism and participates in various intracellular processes, such as cellular signal transduction. AKT is involved in cell cycle regulation, autophagy and apoptosis. mTOR is a crucial signaling molecule that is involved in regulating autophagy, and it is activated by AKT 14, 15. Cellular autophagy is an essential process for maintaining cellular homeostasis, and it can be categorized as selective or nonselective autophagy according to the selectivity with which its substrates are targeted for degradation. Selective autophagy, which mainly includes mitophagy, peroxisomal autophagy, endoplasmic reticulum and ribosomal autophagy, is closely associated with a variety of diseases 16. Mitochondrial damage and dysfunction are involved in the pathogenesis of UC. The morphology of mitochondria is altered via fission and fusion to maintain the stability of mitochondrial dynamics. In the intestinal epithelial cells of UC patients, mitochondrial fission occurs at excessive levels, and mitochondrial fusion is inhibited. UC patients have an increased number of damaged mitochondria, which produce large amounts of reactive oxygen species (ROS). Excessive ROS accumulation in the intestinal mucosa is another important feature of UC 17, 18. Therefore, timely removal of damaged mitochondria is critical for reducing intracellular ROS levels, suppressing inflammation, and maintaining intestinal mucosal homeostasis. Mitophagy is a selective form of autophagy and an extremely important mechanism for maintaining mitochondrial quality. Mitophagy specifically removes damaged mitochondria from cells, maintains homeostasis of mitochondrial dynamics, and reduces ROS production by damaged mitochondria to alleviate cellular damage 19.

Exosomes have been demonstrated to inhibit the PI3K/AKT/mTOR signaling pathway to alleviate UC 20, but the role of mitophagy is unknown. Therefore, this study investigated the therapeutic effects of hypoxia-preconditioned HF-MSC-derived exosomes (Hy-Exos) on UC and examined the differences in Hy-Exos components compared with HF-MSC-derived exosomes (Exos) to further explore the relationship between Hy-Exos and the PI3K/AKT/mTOR signaling pathway and mitophagy.

## Results

### Characterization of HF-MSCs and hypoxia-preconditioned HF-MSCs (Hy-HF-MSCs)

**Figure 1:**
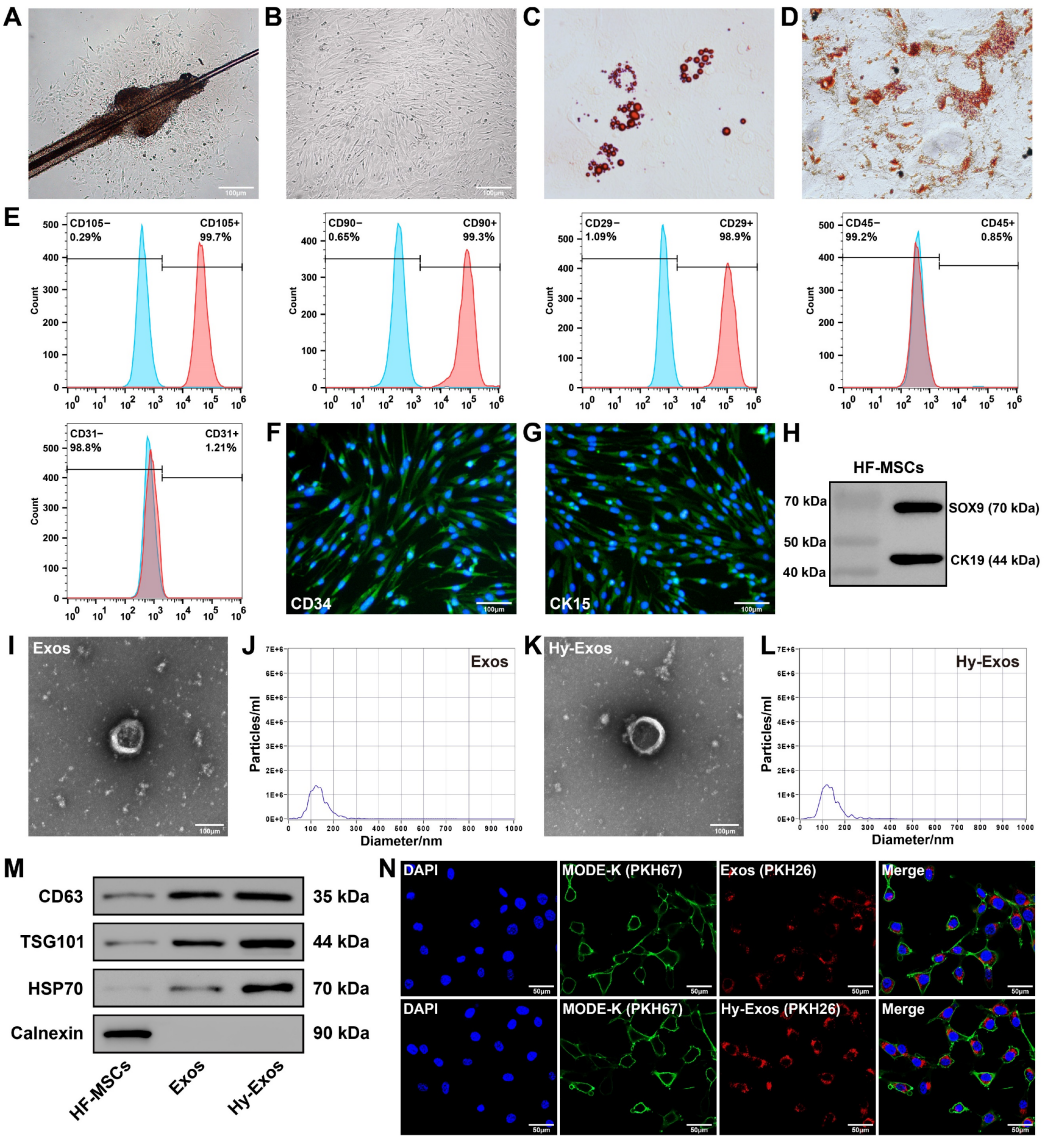
**Characterization of HF-MSCs, Exos and Hy-Exos.** Primary (A) and P3 (B) HF-MSCs. Scale bar = 100 μm. C. Adipogenic differentiation of HF-MSCs. D. Osteogenic differentiation of HF-MSCs. E. Flow cytometry was used to detect HF-MSC-specific antigenic markers. F-G. Immunofluorescence staining were used to measure the expression of CD34 and CK15 in HF-MSCs. Scale bar = 100 μm. H. SOX9 and CK19 expression was measured by western blotting. I. Morphology of Exos by TEM. Scale bar = 100 μm. J. Nanoparticle tracking analysis of Exos. K. Morphology of Hy-Exos by TEM. Scale bar = 100 μm. L. Nanoparticle tracking analysis of Hy-Exos. M. CD63, TSG101, HSP70 and Calnexin expression were measured by western blotting. N. PKH26-labelled Exos and Hy-Exos were internalized by PKH67-labelled MODE-K cells. Scale bar = 50 μm.

The HF-MSCs began to accumulate in the area of the hair follicle bulge and gradually grew around it (Figure 1A). As the cells proliferated, they gradually transformed into a monolayer, and the cells had a long fusiform morphology (Figure 1B). HF-MSCs differentiated into adipocytes and osteocytes (Figure 1C-1D). Most HF-MSCs expressed CD105, CD90 and CD29 (MSC surface markers), and few HF-MSCs expressed CD45 (haematopoietic cell surface marker) or CD31 (endothelial cell surface marker) 21, 22 (Figure 1E). These HF-MSCs were positive for CD34 and CK15 expression according to immunofluorescence staining (Figure 1F-1G) and positive for CK19 and SOX9 expression according to western blotting (Figure 1H), which is consistent with previous findings 23-25. Hy-HF-MSCs exhibited a long fusiform morphology (Figure S1A) and differentiated into adipocytes and osteocytes (Figure S1B-S1C). Most Hy-HF-MSCs expressed CD105, CD90 and CD29, and few Hy-HF-MSCs expressed CD45 or CD31 (Figure S1D).

### Characterization of Exos and Hy-Exos

The Exos had a saucer-like shape, with diameters that were mostly between 50 and 200 nm (Figure 1I-1J). The morphology and diameter of the Hy-Exos were not significantly different from those of the Exos (Figure 1K-1L). Western blotting analysis revealed positive expression of the Exos and Hy-Exos surface marker proteins CD63, TSG101 and HSP70, and negative expression of Calnexin (Figure 1M). PKH26-stained Exos and Hy-Exos were taken up by PKH67-stained MODE-K cells (Figure 1N).

### Hy-Exos promote LPS-induced MODE-K cells recovery

**Figure 2:**
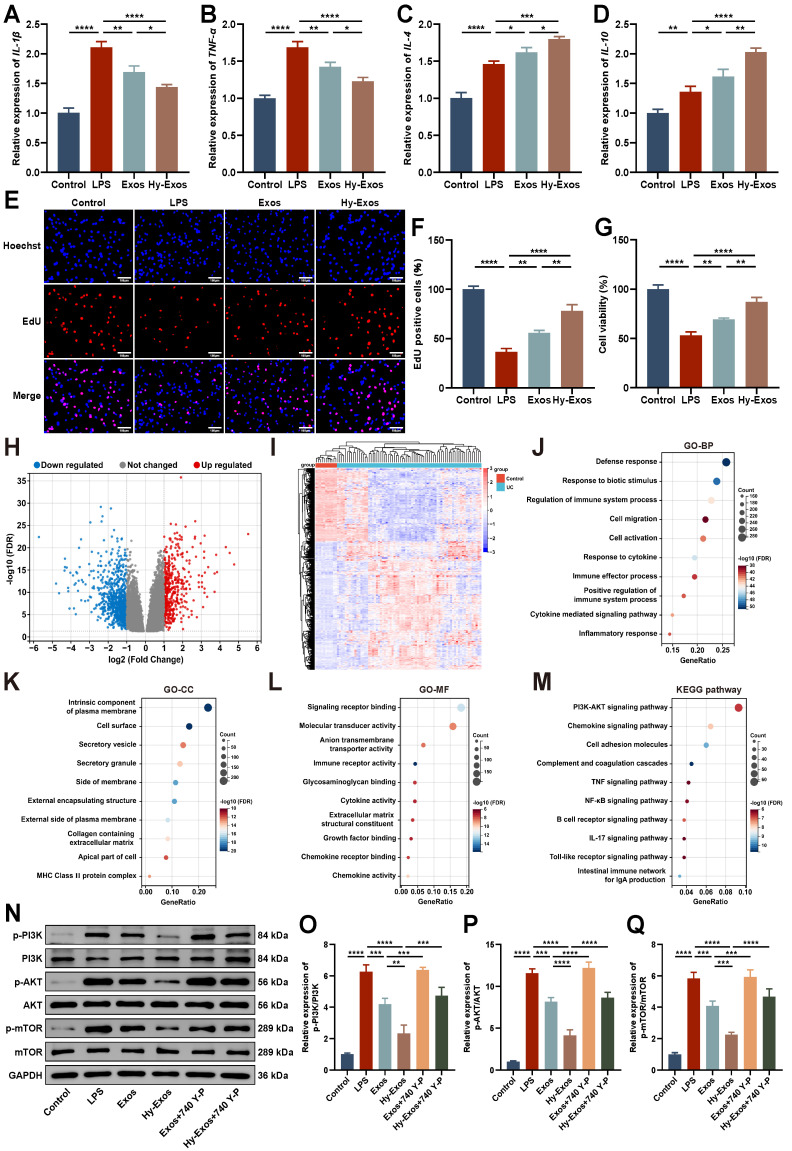
**Hy-Exos inhibit the PI3K/AKT/mTOR signaling pathway and promote LPS-induced MODE-K cells recovery.** A-D. *IL-1β*, *TNF-α*, *IL-4* and *IL-10* expression in MODE-K cells was measured by PCR. E-F. MODE-K cells proliferation in each group was determined by EdU staining. Scale bar = 100 μm. G. MODE-K cells viability in each group was detected by CCK-8 assays. H. Volcano plot of DEGs in GSE75214. I. Heat map of DEGs in GSE75214. J-L. GO enrichment analysis of DEGs. M. KEGG enrichment analysis of DEGs. N-Q. Western blotting was used to measure p-PI3K/PI3K, p-AKT/AKT and p-mTOR/mTOR expression in MODE-K cells. Data are shown as the mean ± SD. (*p < 0.05, **p < 0.01, ***p < 0.001, ****p < 0.0001).

LPS (1 μg/mL)-treated MODE-K cells were used to successfully establish a model of inflammation. Hy-Exos reduced *IL-1β* and *TNF-α* expression, and increased *IL-4* and *IL-10* expression in LPS-treated MODE-K cells (Figure 2A-2D). EdU staining and Cell Counting Kit-8 (CCK-8) assays showed that the proliferation and viability of MODE-K cells decreased after LPS treatment, but the administration of Hy-Exos reversed this effect (Figure 2E-2G).

### Hy-Exos inhibit the PI3K/AKT/mTOR signaling pathway

The GSE75214 dataset was downloaded from the GEO database, and it contained 194 samples, including 74 active UC tissues, 23 inactive UC tissues, 8 Crohn's disease tissues, 11 control tissues, and terminal ileum tissues from 67 CDs and 11 controls. This dataset included samples from UC patients in different disease states. This study focused on 74 active UC tissues and 11 control tissues in this dataset. The differentially expressed genes (DEGs) between the UC group and control group were determined using the GEO2R tool. A total of 1265 DEGs were identified. Compared with the control group, 454 genes were upregulated and 811 genes were downregulated in the UC group. Volcano plot and heat map show the identified DEGs (Figure 2H-2I). Gene Ontology (GO) enrichment analysis mainly included three main categories: biological process (BP), cellular component (CC) and molecular function (MF) (Figure 2J-2L). Kyoto Encyclopedia of Genes and Genomes (KEGG) enrichment analyses revealed that the DEGs were primarily correlated with the PI3K-AKT signaling pathway, chemokine signaling pathway and cell adhesion molecules (Figure 2M). The enrichment results of DEGs in the PI3K-AKT signaling pathway were shown in detail in Figure S2. Western blotting demonstrated that p-PI3K/PI3K, p-AKT/AKT, and p-mTOR/mTOR expression were increased after LPS treatment, decreased after Hy-Exos treatment, and then increased again after the addition of 740 Y-P (Figure 2N-2Q). These findings suggested that Hy-Exos inhibited the PI3K/AKT/mTOR signaling pathway.

### Hy-Exos maintain mitochondrial dynamic stabilization, alleviate mitochondrial dysfunction and enhance autophagy in MODE-K cells

**Figure 3:**
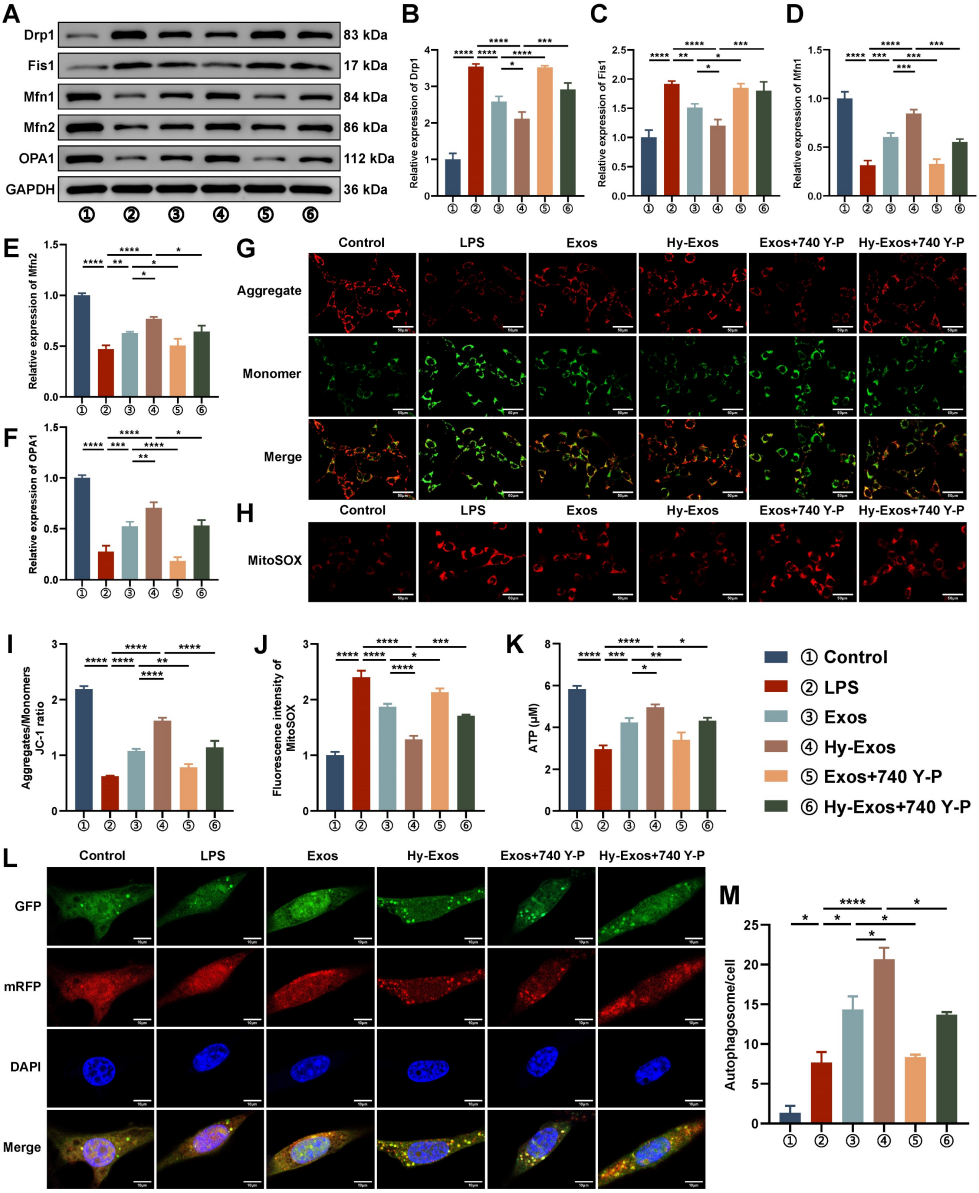
** Hy-Exos maintain mitochondrial dynamic stabilization, alleviate mitochondrial dysfunction and enhance autophagy in MODE-K cells.** A-F. Western blotting was used to measure Drp1, Fis1, Mfn1, Mfn2 and OPA1 expression in MODE-K cells. G, I. Mitochondrial membrane potential in MODE-K cells as measured by a JC-1 staining kit, and the ratio of aggregates (red) to monomers (green) was calculated. Scale bar = 50 μm. H, J. Mitochondrial ROS was detected in MODE-K cells. Scale bar = 50 μm. K. Determination of ATP contents in MODE-K cells. L-M. MODE-K cells were infected with mRFP-EGFP-LC3 adenovirus, and the number of yellow spots (autophagosomes) was observed under a microscope. Scale bar = 10 μm. Data are shown as the mean ± SD. (*p < 0.05, **p < 0.01, ***p < 0.001, ****p < 0.0001).

In LPS-treated MODE-K cells, Drp1 and Fis1 were increased, and Mfn1, Mfn2 and OPA1 were decreased. The administration of Hy-Exos reversed these changes (Figure 3A-3F). The mitochondrial membrane potential was determined with a JC-1 staining kit. LPS-treated MODE-K cells showed an increase in the number of monomers (green fluorescence) and a decrease in the number of aggregates (red fluorescence), and the administration of Hy-Exos had the opposite effect (Figure 3G, 3I). After LPS treatment, ROS production by mitochondria increased, and the administration of Hy-Exos reversed this effect (Figure 3H, 3J). In addition, the intracellular ATP content was decreased by LPS treatment and increased after the administration of Hy-Exos (Figure 3K). Compared with LPS treatment, MODE-K cells treated with the PI3K inhibitor LY294002 exhibited decreased numbers of monomers, increased numbers of aggregates, and decreased levels of ROS in mitochondria (Figure S3A-S3B). Taken together, Hy-Exos alleviated mitochondrial dysfunction in MODE-K cells. Moreover, compared with the LPS group, yellow fluorescence in the Hy-Exos group was significantly increased in the mRFP-EGFP-LC3 adenovirus-infected MODE-K cells (Figure 3L). The strength of the autophagic flux was determined by counting the number of yellow spots in each group, and the results were statistically analysed (Figure 3M).

### Hy-Exos enhance mitophagy in MODE-K cells

**Figure 4:**
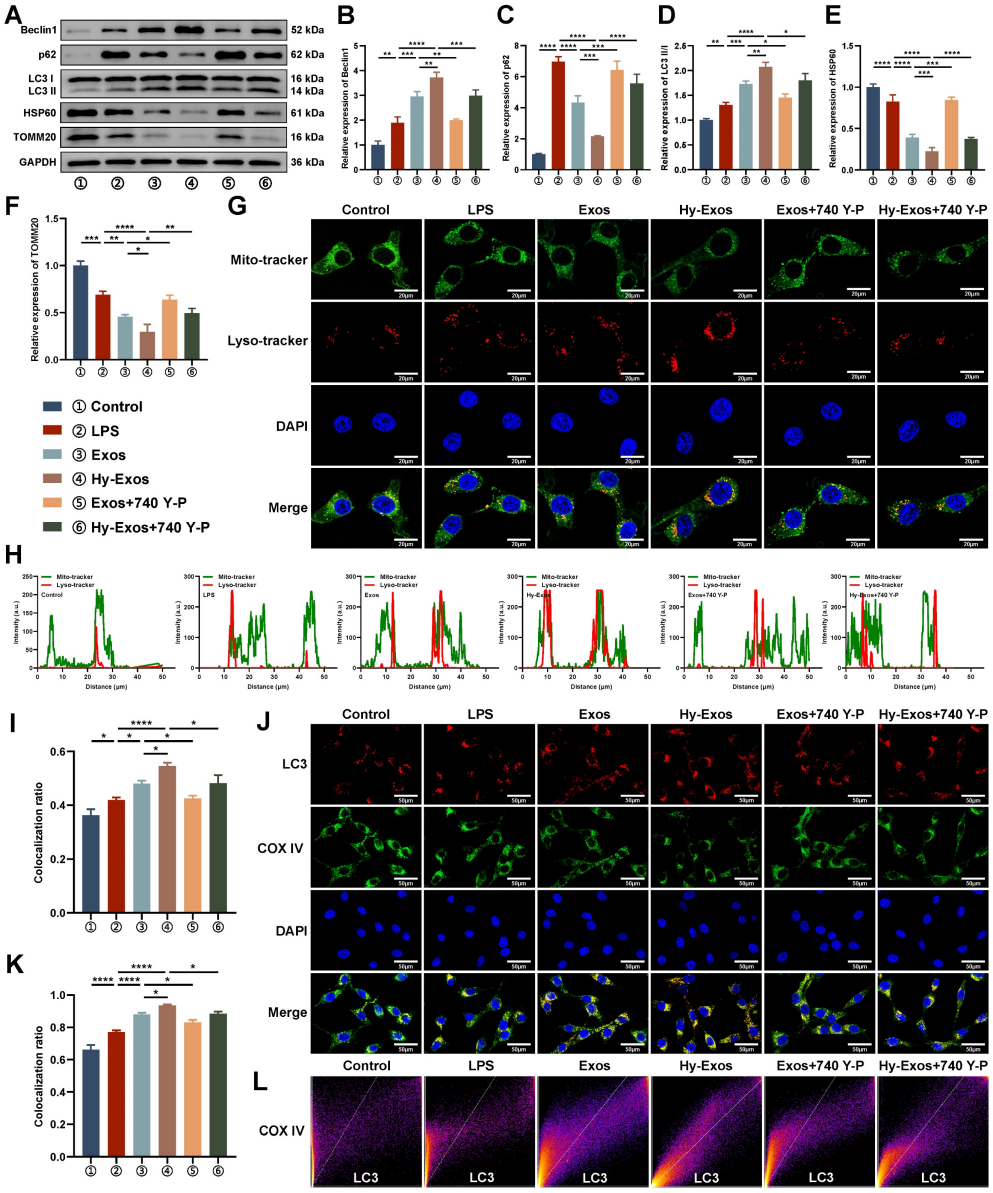
**Hy-Exos enhance mitophagy in MODE-K cells.** A-F. Beclin1, p62, LC3II/I, HSP60 and TOMM20 expression in MODE-K cells was measured by western blotting. G. Mitochondrial probe and lysosome probe staining in MODE-K cells. Scale bar = 20 μm. H-I. Analysis of the colocalization of the mitochondrial probe and lysosomal probe. J. Immunofluorescence staining for LC3 and COX IV in MODE-K cells. Scale bar = 50 μm. K-L. The colocalization of LC3 and COX IV analysis. Data are shown as the mean ± SD. (*p < 0.05, **p < 0.01, ***p < 0.001, ****p < 0.0001).

Hy-Exos reversed the LPS-induced decreases in Beclin1 and LC3II/I expression and increases in p62, HSP60 and TOMM20 expression in MODE-K cells (Figure 4A-4F). MODE-K cells were stained with a mitochondrial probe and a lysosome probe. The results demonstrated that Hy-Exos enhanced the colocalization of mitochondria and lysosomes in MODE-K cells compared with LPS treatment (Figure 4G). We further analysed the colocalization results (Figure 4H-4I). Compared with LPS treatment, Hy-Exos increased LC3 expression, and promoted the colocalization of LC3 and COX IV in MODE-K cells, as shown by immunofluorescence staining (Figure 4J-4L).

### Differentially expressed miRNAs (DEMs) identification

**Figure 5:**
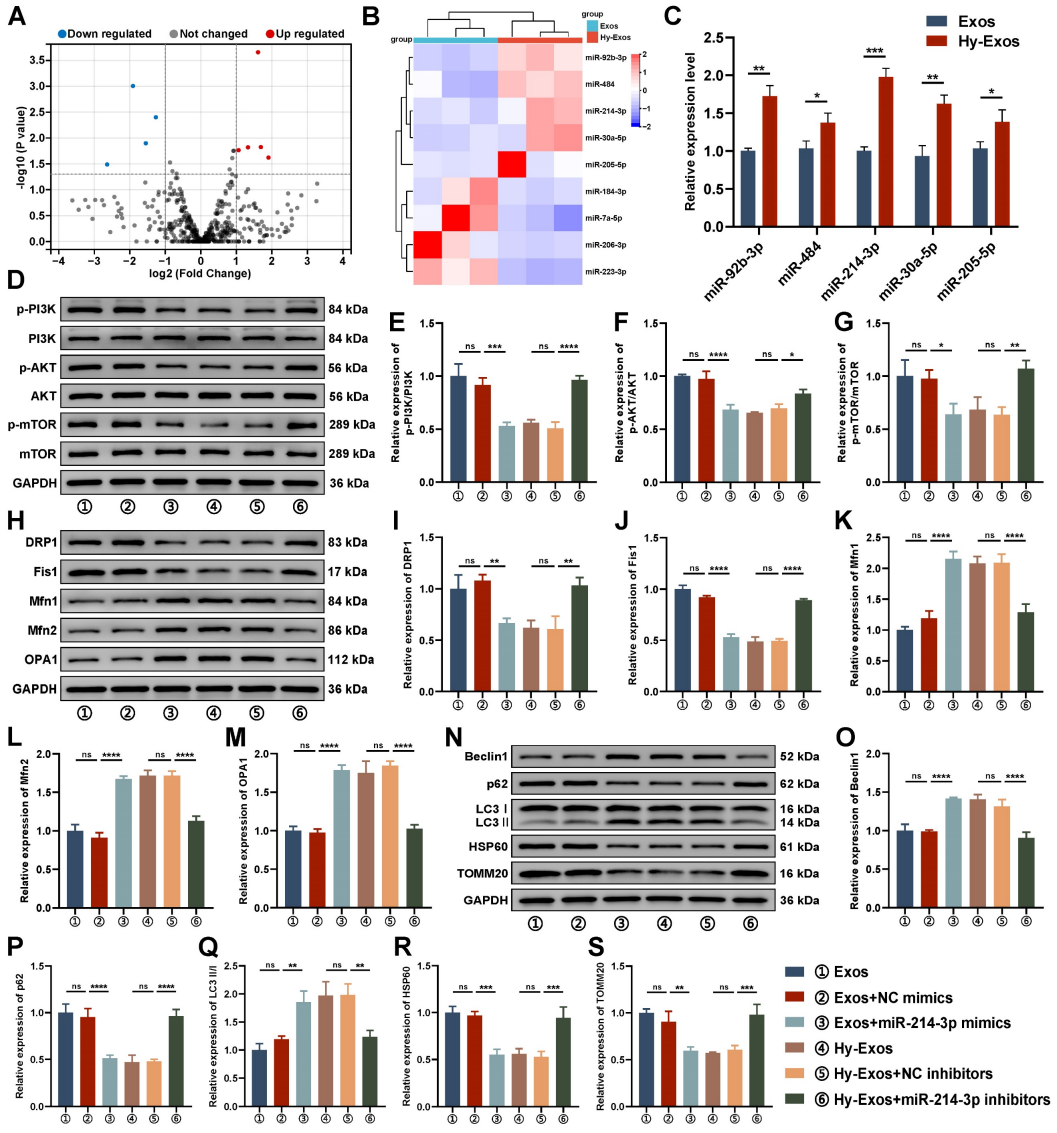
** DEMs identification and the role of miR-214-3p in Hy-Exos.** A. Volcano plot of DEMs. B. Heat map of DEMs. C. PCR was used to detect the expression of miR-92b-3p, miR-484, miR-214-3p, miR-30a-5p and miR-205-5p in the Exos and Hy-Exos groups. D-G. Western blotting was used to measure p-PI3K/PI3K, p-AKT/AKT and p-mTOR/mTOR expression in MODE-K cells. H-M. Western blotting was used to measure Drp1, Fis1, Mfn1, Mfn2 and OPA1 expression in MODE-K cells. N-S. Western blotting was used to measure Beclin1, p62, LC3II/I, HSP60 and TOMM20 expression in MODE-K cells. Data are shown as the mean ± SD. (*p < 0.05, **p < 0.01, ***p < 0.001, ****p < 0.0001).

To investigate the differences in composition between Exos and Hy-Exos, miRNA sequencing was performed. Compared with Exos, five miRNAs were upregulated, and four miRNAs were downregulated in Hy-Exos. Volcano plot and heat map show the DEMs (Figure 5A-5B). PCR was used to detect the expression of miR-92b-3p, miR-484, miR-214-3p, miR-30a-5p and miR-205-5p in the Exos and Hy-Exos groups, and the results showed that the expression of miR-214-3p was the most significantly different (Figure 5C).

### The role of miR-214-3p in Hy-Exos

Compared with the Exos + NC mimics group, p-PI3K/PI3K, p-AKT/AKT and p-mTOR/mTOR expression were decreased in the Exos + miR-214-3p mimics group. Compared with the Hy-Exos + NC inhibitors group, p-PI3K/PI3K, p-AKT/AKT and p-mTOR/mTOR expression were increased in the Hy-Exos + miR-214-3p inhibitors group (Figure 5D-5G). In addition, mitochondrial fission was decreased, fusion was increased, and mitophagy was increased in the Exos + miR-214-3p mimics group compared with the Exos + NC mimics group. Compared with the Hy-Exos + NC inhibitors group, mitochondrial fission was increased, mitochondrial fusion was decreased, and mitophagy was decreased in the Hy-Exos + miR-214-3p inhibitors group (Figure 5H-5S).

### *Ex vivo* organ distribution of Exos and Hy-Exos in mice

**Figure 6:**
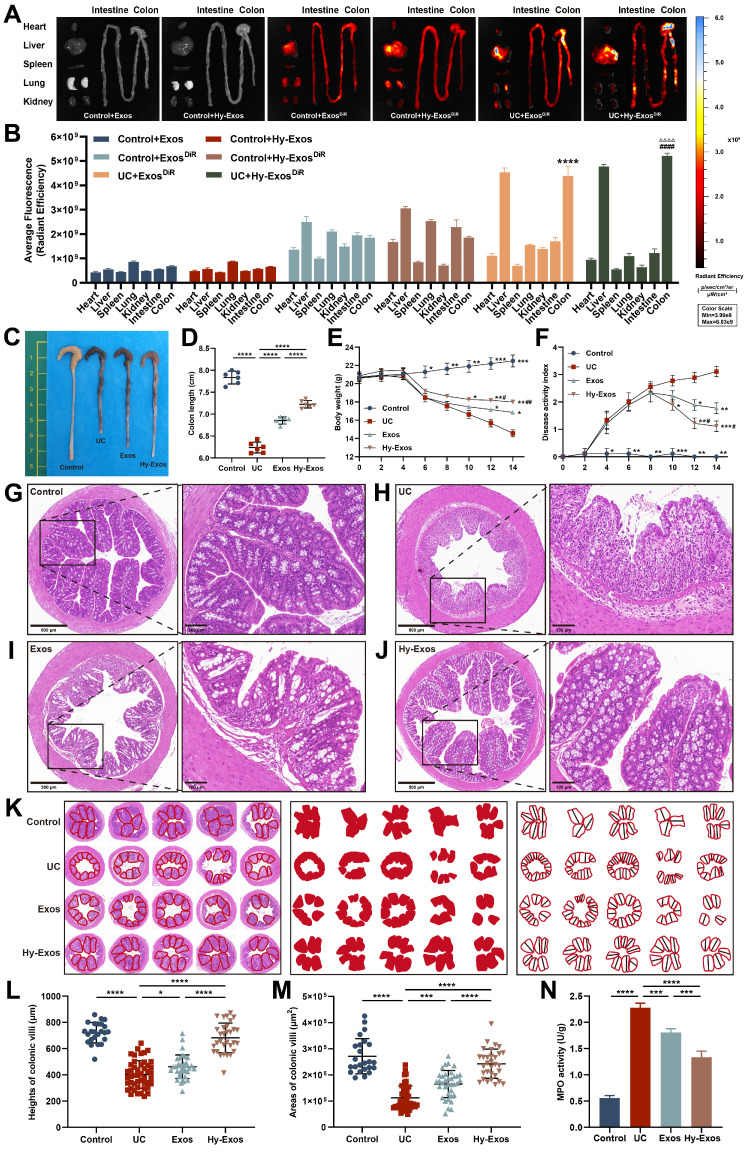
**Hy-Exos promote the amelioration of DSS-induced UC.** A-B. Mice in the control group and UC group were given DiR-stained Exos and Hy-Exos via the tail vein. The mice were subjected to ex vitro organ imaging and quantitative analysis of fluorescence intensity. C-D. Comparison of the colon lengths of the mice in each group. E. Comparison of body weight changes in mice in each group. F. DAI scores were determined in mice by monitoring changes in body weight, faecal traits, and the degree of haematochezia. G-J. HE staining results of mice in each group. Scale bar = 500 μm (left). Scale bar = 100 μm (right). K-M. The area and height of the colonic villi of mice in each group were measured and analysed. N. MPO expression in colonic tissue homogenates from mice in each group was determined. Data are shown as the mean ± SD. (*p < 0.05, **p < 0.01, ***p < 0.001, ****p < 0.0001,^ #^*p* < 0.05, ^##^*p* < 0.01, ^####^*p* < 0.0001, ^△△△△^*p* < 0.0001).

To determine whether Exos and Hy-Exos reached the sites of colon injury, Exos and Hy-Exos were stained with DiR and observed 12 h after tail vein injection. The results showed that the stained Exos and Hy-Exos were slightly distributed in all organs of normal mice, while were mainly concentrated in the liver and colon of UC mice, and the fluorescence signal of Hy-Exos in the colon was stronger than that of Exos (Figure 6A-B).

### Hy-Exos promote the amelioration of dextran sulfate sodium (DSS)-induced UC

The length of the colon was significantly shortened in mice that were induced by DSS, and the length of the colon was slightly restored after Hy-Exos treatment (Figure 6C-6D). After DSS induction, the body weights of the mice gradually decreased, and the DAI scores gradually increased. After Hy-Exos treatment, mice body weights loss were inhibited, and the DAI scores gradually decreased (Figure 6E-6F). Colon tissues from mice in each group were stained with HE. In the control group, the colonic mucosa was intact, and in the UC group, most of the colonic mucosa was damaged, with absent glands and inflammatory cell infiltration. The Exos and Hy-Exos groups exhibited amelioration of colonic mucosal damage, with the Hy-Exos group exhibited greater improvement in mucosal damage and less inflammatory cells infiltration (Figure 6G-6J). The villus heights and areas in mice colon tissues were measured. In the UC group, the villus heights and areas were reduced, and the administration of Hy-Exos reversed these changes (Figure 6K-6M). Moreover, the myeloperoxidase (MPO) content in colon tissue homogenates was elevated in the UC group, and this increase was reversed by Hy-Exos treatment (Figure 6N).

### Hy-Exos inhibit the PI3K/AKT/mTOR signaling pathway, maintain mitochondrial dynamic stabilization and alleviate mitochondrial dysfunction in UC mice

**Figure 7:**
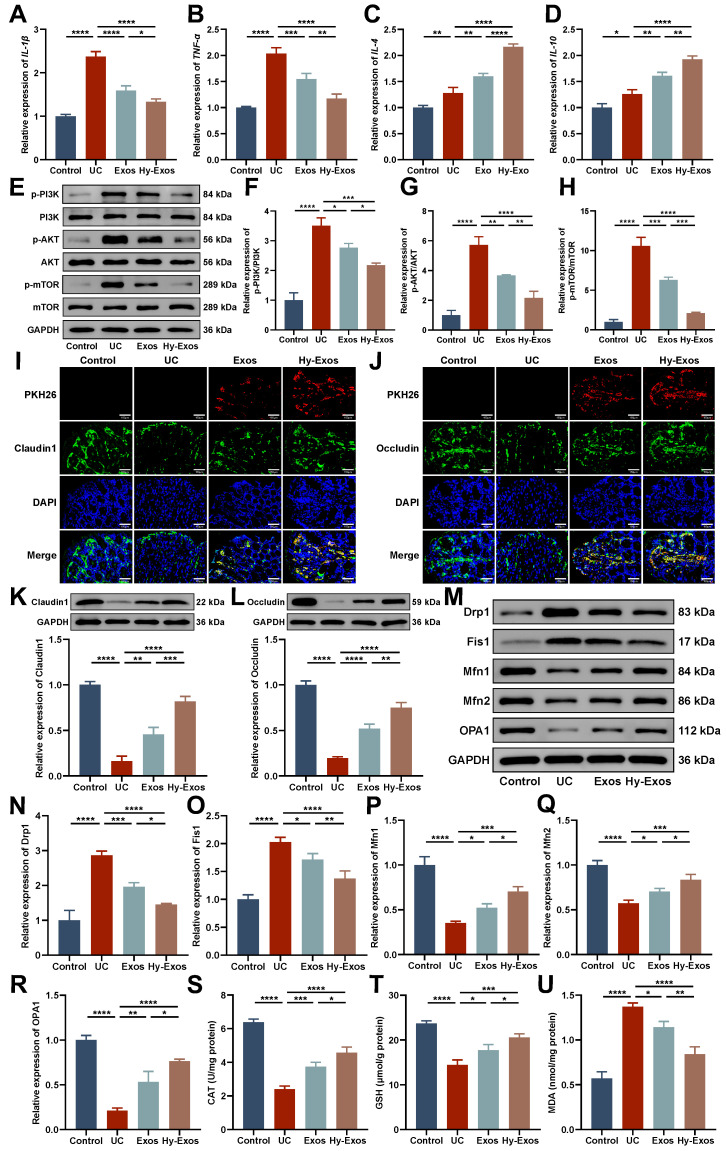
** Hy-Exos inhibit the PI3K/AKT/mTOR signaling pathway, maintain mitochondrial dynamic stabilization and alleviate mitochondrial dysfunction in UC mice.** A-D. RT-PCR was performed to measure *IL-1β*, *TNF-α*, *IL-4* and *IL-10* expression in the colon tissues of mice. E-H. Western blotting was used to measure p-PI3K/PI3K, p-AKT/AKT and p-mTOR/mTOR expression in colon tissues. I-J. PKH26-stained Exos and Hy-Exos colocalized with Claudin1 and Occludin. Scale bar = 150 μm. K-L. Claudin1 and Occludin expression in colon tissues was determined by western blotting. M-R. Western blotting was used to measure the expression of Drp1, Fis1, Mfn1, Mfn2, and OPA1 in colon tissues. S-U. Determination of the CAT, GSH, and MDA contents in colon tissues. Data are shown as the mean ± SD. (*p < 0.05, **p < 0.01, ***p < 0.001, ****p < 0.0001).

PCR results showed that Hy-Exos reduced *IL-1β* and *TNF-α* expression and increased *IL-4* and *IL-10* expression compared with the UC group (Figure 7A-7D). Western blotting demonstrated that p-PI3K/PI3K, p-AKT/AKT, and p-mTOR/mTOR expression were increased in the UC group and decreased after Hy-Exos treatment (Figure 7E-7H). Immunofluorescence staining revealed that PKH26-stained Exos and Hy-Exos reached the intestinal injury site and promoted the expression of Claudin1 and Occludin in UC mice. Hy-Exos accumulated at the intestinal injury site in higher amounts and more strongly promoted the restoration of tight junctions (Figure 7I-7J). Western blotting analysis showed the same results (Figure 7K-7L). In addition, Drp1 and Fis1 were decreased, and Mfn1, Mfn2 and OPA1 were increased in the Hy-Exos group compared with the UC group (Figure 7M-7R). Moreover, antioxidant defence is also one of the major functions of mitochondria. Compared with the UC group, Hy-Exos treatment increased catalase (CAT) and glutathione (GSH) expression and decreased the expression of malondialdehyde (MDA) (Figure 7S-7U).

### Hy-Exos enhance mitophagy in UC mice

**Figure 8:**
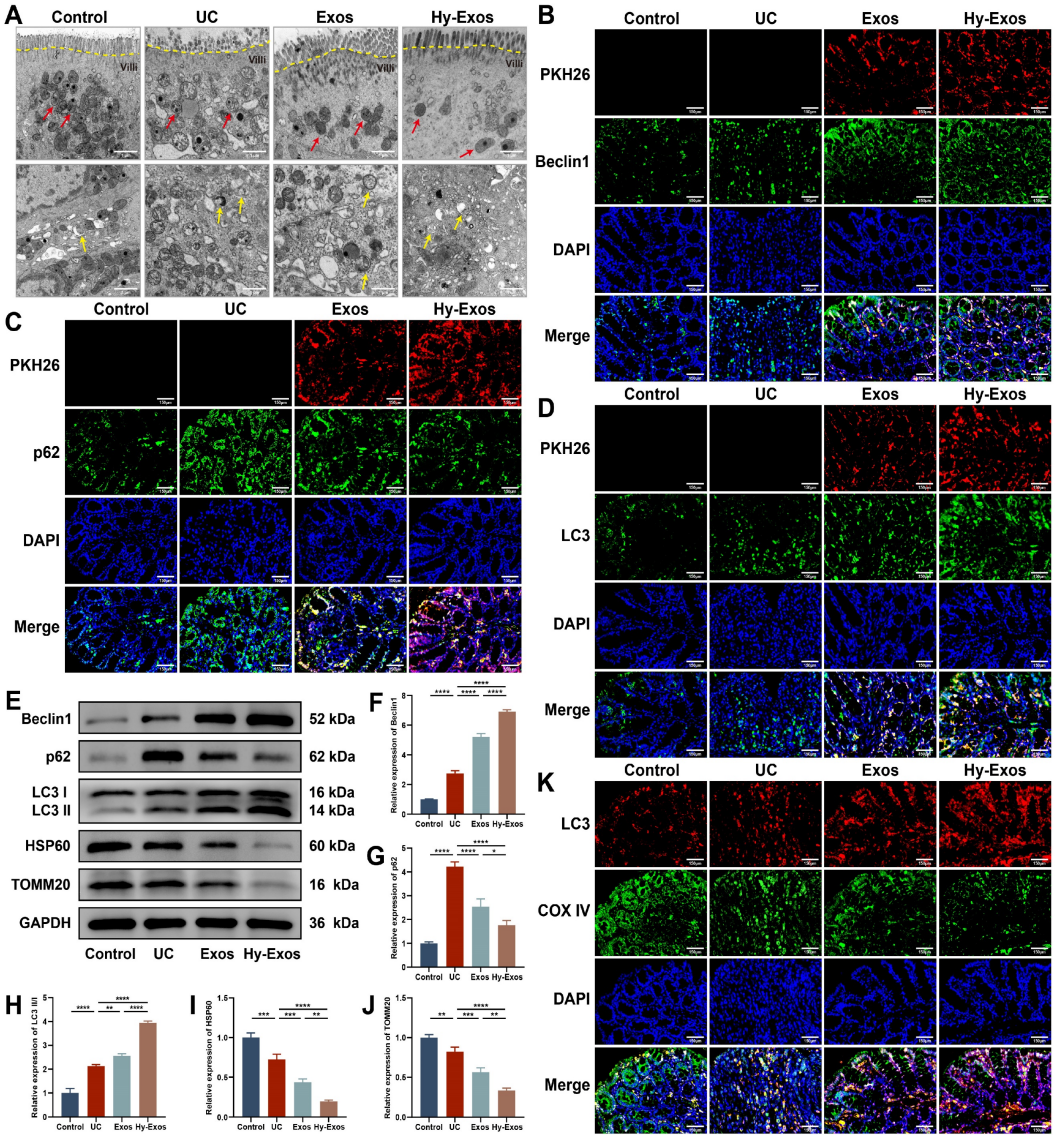
**Hy-Exos enhance mitophagy in UC mice.** A. TEM was performed to observe the ultrastructure of the colonic tissues of mice, and the formation of mitochondria (red arrows) and autophagosomes (yellow arrows) was observed. Scale bar = 1 μm. B-D. PKH26-stained Exos and Hy-Exos colocalized with Beclin1, p62 and LC3II/I. Scale bar = 150 μm. E-J. Western blotting was used to measure Beclin1, p62, LC3II/I, HSP60 and TOMM20 expression in colon tissues. K. Immunofluorescence staining for LC3 and COX IV in colon tissues. Scale bar = 150 μm. Data are shown as the mean ± SD. (*p < 0.05, **p < 0.01, ***p < 0.001, ****p < 0.0001).

Transmission electron microscopy (TEM) revealed that the mitochondria had a normal morphology in the control group, and the mitochondrial cristae were structurally intact and neatly arranged. In the UC group, the mitochondrial contents were discharged, the mitochondrial cristae were shortened and shifted sideways, the matrix particles were reduced, and cavitation was observed. The administration of Exos and Hy-Exos ameliorated mitochondrial damage, and more autophagosome formation was observed in the Hy-Exos group (Figure 8A). Immunofluorescence staining showed that PKH26-stained Exos and Hy-Exos colocalized with Beclin1, p62 and LC3II/I, promoted Beclin1 and LC3II/I expression, and inhibited p62 expression, and these effects were stronger in the Hy-Exos group compared with the UC group (Figure 8B-8D). Furthermore, western blotting analysis showed the same results, and Hy-Exos inhibited the expression of HSP60 and TOMM20 (Figure 8E-8J). In addition, Hy-Exos increased LC3 expression and promoted the colocalization of LC3 with COX IV compared with the UC group (Figure 8K).

## Discussion

UC is an inflammatory bowel disease for which effective therapeutic strategies are lacking. PI3K/AKT/mTOR signaling pathway activation and mitochondrial damage play crucial roles in the development of UC. This study compared the differences in components between Exos and Hy-Exos and innovatively proposed that Hy-Exos maintain mitochondrial dynamic stabilization, alleviate mitochondrial dysfunction and enhance mitophagy to ameliorate UC by inhibiting the PI3K/AKT/mTOR signaling pathway via miR-214-3p.

In recent years, cell therapy has been used to treat a variety of diseases. Bone marrow mesenchymal stem cells (BMSCs) have been widely studied, but the damage caused by their extraction and the small number of applications limit their clinical application. HF-MSCs are abundant, easily accessible with little harm to donors, and have greater proliferative capacity than BMSCs 26. However, the transplantation of HF-MSCs remains somewhat controversial because of their low survival rate after transplantation 27. Studies have demonstrated that the therapeutic effect of MSC transplantation mainly occurs via the paracrine effects of exosomes.

Under different stimulation environments, exosomes released from MSCs perform different functions and participate in different molecular regulatory mechanisms to affect regeneration and repair. Under hypoxic conditions, exosomes released from MSCs exert stronger effects than normal exosomes 28, 29. In addition, compared with 3D culture, scaffold material engineering and other methods, hypoxia preconditioning is easier to achieve and can be used for large-scale production. Therefore, in this study, Hy-Exos were extracted, and LPS-treated MODE-K cells were found to take up Hy-Exos, which promoted MODE-K cells viability and proliferation recovery while decreasing the expression of pro-inflammatory factors and increasing the expression of anti-inflammatory factors. DSS was administered to establish a mouse UC model, which exhibited weight loss, bloody stools, increased DAI scores, shortened colon lengths and inflammatory changes that were visible by HE staining. In addition, MPO is an indicator of the degree of neutrophil infiltration, which was increased in the UC group. These changes indicate that the UC model was successfully established. The administration of Hy-Exos reversed these changes, which suggests that Hy-Exos alleviate UC by reducing the inflammatory response.

The severity of UC is related to the severity of intestinal mucosal epithelial barrier damage. Tight junctions are the most important type of intercellular junctions that maintain the integrity of the intestinal mucosal epithelial barrier. Claudin1 and Occludin are two tight junction proteins that play critical roles in maintaining epithelial barrier function in the intestinal mucosa by regulating intercellular material flow, maintaining epithelial cell polarity, and blocking the passage of toxic macromolecules and microorganisms 30. PKH26-stained Exos and Hy-Exos accumulated in the colon tissues of UC mice, and they colocalized with Claudin1 and Occludin. Compared with the control group, Claudin1 and Occludin expression decreased in the UC group, whereas Claudin1 and Occludin expression increased after Hy-Exos treatment. This result indicated that Hy-Exos promoted intestinal mucosal epithelial barrier recovery.

The PI3K/AKT/mTOR signaling pathway is one of the most commonly explored pathways in targeted therapy. Bioinformatics technology was used to identify DEGs between the control group and UC group in the GSE75214 dataset of the GEO database. KEGG enrichment analyses of the DEGs demonstrated that they were closely linked to the PI3K/AKT/mTOR signaling pathway. This pathway regulates oxidative stress and inflammation development, and it is involved in the regulation of autophagy in mammals 31. Studies have shown that inhibition of mTOR alleviates mitochondrial dysfunction and enhances mitophagy 32. This study demonstrated that the PI3K/AKT/mTOR signaling pathway was upregulated in LPS-treated MODE-K cells and colon tissues from UC mice, and Hy-Exos reversed this change. The administration of the PI3K activator 740Y-P further verified the role of Hy-Exos.

Mitochondria are important organelles that provide energy and regulate cell metabolism. Mitochondrial dynamics refer to the continuous fission and fusion of mitochondria that are regulated by related proteins to maintain structural stability and intracellular environmental balance, which results in changes in quality and morphology 33. Drp1 and Fis1 are mitochondrial fission-associated proteins, and Mfn1, Mfn2 and OPA1 are mitochondrial fusion-associated proteins that are involved in the development of UC and are associated with intestinal barrier function and epithelial vitality 18, 34. This study revealed that Drp1 and Fis1 were increased, and Mfn1, Mfn2 and OPA1 were decreased in the LPS-treated group, and these changes could be reversed by the administration of Hy-Exos. Similar results were obtained in mice. These results indicate that excessive mitochondrial fission promotes the occurrence and development of diseases, and mitochondrial fusion promotes mitochondrial self-repair and inhibits disease progression. Hy-Exos inhibited mitochondrial fission, promoted mitochondrial fusion, and maintained mitochondrial dynamic stabilization.

Mitochondria also play important roles in cellular oxidative metabolism. Inflammation leads to the production of large amounts of intracellular ROS, which causes changes in mitochondrial structure and results in mitochondrial dysfunction, organelle swelling, and ultimately cell death. ROS are produced primarily by damaged mitochondria, which deteriorates the intracellular environment and exacerbates cell damage 35, 36. This study revealed that MODE-K cells treated with LPS exhibited decreased mitochondrial membrane potential, excessive production of mitochondrial ROS, and decreased ATP content. The mitochondrial membrane potential reflects the functional state of mitochondria. ROS cause a loss of mitochondrial endomembrane permeability, which results in a reduction in the mitochondrial membrane potential. Mitochondria are the powerhouses of the cell and serve as the source of ATP to maintain a variety of cellular physiological processes. Induction with LPS can result in mitochondrial dysfunction and insufficient ATP production in MODE-K cells, and the administration of Hy-Exos can alleviate mitochondrial dysfunction. In UC mice, colon tissues exhibited decreased CAT and GSH expression, and increased MDA expression, and these effects were reversed by the administration of Hy-Exos. CAT scavenges hydrogen peroxide and prevents oxidative stress in the body. GSH is involved in clearing ROS and preventing oxidative stress from damaging the body. MDA reflects the level of lipid peroxidation damage 37, 38. These results indicate that Hy-Exos have a certain antioxidant capacity. Moreover, after infection of MODE-K cells with mRFP-EGFP-LC3 adenovirus, yellow fluorescence was enhanced in the Hy-Exos group compared with LPS group, which indicated that Hy-Exos affected autophagic flux and enhanced the autophagic activity of these MODE-K cells. These results showed that the amelioration of mitochondrial dysfunction was accompanied by autophagy.

The colon tissues of UC mice exhibited severe mitochondrial damage and a small number of autophagosomes according to TEM. After the administration of Hy-Exos, a large number of autophagosomes formed. This study also showed that Hy-Exos increased Beclin1 and LC3II/I expression and decreased p62, TOMM20 and HSP60 expression in LPS-treated MODE-K cells and colon tissues of UC mice. Beclin1 is a key protein that regulates the initiation of autophagy, and it participates in the formation of autophagosomes in the early stage of autophagy 39. LC3 is an essential protein for inducing autophagosome formation. When mitochondrial autophagy is initiated, p62 mediates the binding of LC3 to ubiquitinated proteins on the mitochondrial outer membrane to promote the fusion of mitochondria and autophagosomes, which are subsequently degraded by autophagolysosomes 16, 40. TOMM20 and HSP60 are marker proteins of the mitochondrial outer membrane and mitochondrial matrix, respectively 41. Since mitophagy is a process of mitochondrial biodegradation, TOMM20 and HSP60 expression negatively correlates with mitophagic activity. In addition, PKH26-stained Exos and Hy-Exos colocalized with Beclin1, p62 and LC3 in the colon tissues of UC mice. And Hy-Exos enhanced Beclin1 and LC3II/I expression and reduced p62 expression more significantly. Similar results were observed at the protein level, and Hy-Exos also decreased the expression of TOMM20 and HSP60. These findings suggested that Hy-Exos enhanced mitophagy both *in vitro* and* in vivo*.

Additionally, Hy-Exos promoted the colocalization of mitochondria and lysosomes and of LC3 and COX IV in MODE-K cells. Lysosomes, which are essential organelles in autophagy, regulate mTOR signals to initiate autophagosome formation and substrate degradation 42, 43. COX IV is one of the cytochrome C oxidase subunits, which can be used as a marker of mitochondria 44. These results suggested that Hy-Exos promoted the fusion of mitochondria and autophagosomes and enhanced mitophagy. Similarly, Hy-Exos promoted LC3 and COX IV colocalization in the colon tissues of UC mice. In conclusion, Hy-Exos may alleviate UC by enhancing mitophagy.

Hypoxia stimulates the further secretion of cellular exosomes and alters the composition and content of RNA and proteins in exosomes 45. MiRNAs are relatively stable in exosomes and are not susceptible to RNase degradation. Exosomes mainly regulate recipient cells function by delivering miRNAs and are involved in the pathological process of disease 46-48. The miRNA sequencing results indicated that miR-214-3p was the most significantly upregulated in Hy-Exos. This study further investigated the effects of Exos + miR-214-3p mimics and Hy-Exos + miR-214-3p inhibitors on LPS-treated MODE-K cells. The results showed that miR-214-3p was essential for Hy-Exos-mediated inhibition of the PI3K/AKT/mTOR signaling pathway, maintenance of mitochondrial dynamic stability, alleviation of mitochondrial dysfunction and enhancement of mitophagy.

Studies have shown that Hy-Exos inhibit ROS accumulation and DNA damage in intestinal epithelial cells, regulate the balance of macrophages, and promote M2 macrophage polarization, which inhibit the progression of UC 49, 50. In addition, increasing evidence suggests a close link between the gut microbiota and UC. Various bacterial communities and certain metabolites within the gut play a collective role in maintaining intestinal homeostasis. The gut microbiota engage in intercellular communication via the release of exosomes, which can be involved in immunomodulatory processes in UC 51, 52. The aim of this study was to provide complementary insight into the potential mechanism of action of Hy-Exos in ameliorating UC and to provide a new idea for UC treatment. However, this study also has several limitations, such as a short observation time and uncertain long-term effects. There are currently few reports on the clinical use of Hy-Exos in the treatment of UC, and in the future, we will focus on exploring the applicability and potential of Hy-Exos in the process of clinical translation.

In conclusion, Hy-Exos had a good therapeutic effect on LPS-treated MODE-K cells and mice with DSS-induced UC. Hy-Exos promoted colonic tight junction proteins expression, suppressed the oxidative stress response, and reduced UC-related inflammatory injury. Hy-Exos may exert these effects via miR-214-3p-mediated inhibition of the PI3K/AKT/mTOR signaling pathway, maintenance of mitochondrial dynamic stability, alleviation of mitochondrial dysfunction and enhancement of mitophagy.

## Methods

### DEGs identification and enrichment analysis

The GSE75214 dataset was downloaded from the GEO database and generated by the GPL6244 platform. GEO2R was used to preprocess the data and screen the DEGs between the UC and control groups. Adjusted *P* < 0.05 and |log2-fold change| >1 were used as thresholds for each group. The DEGs were subjected to enrichment analysis by GO and KEGG analyses. Differences in enrichment were considered significant when *P* < 0.05.

### Experimental animals

4-6 weeks C57BL/6J mice were selected to establish the UC model, and 7-10 days mice were selected for HF-MSCs extraction. The mice were obtained from the Animal Center of the Second Affiliated Hospital of Harbin Medical University and maintained a 12 h light/dark cycle. All the experiments and methods were approved by the Second Affiliated Hospital of Harbin Medical University Ethics Committee (No. SYDW2023-054).

### Cell preparation

Mouse skin around the vibrissae was removed and cut into small pieces perpendicular to the skin surface. Tissue blocks were digested at 37 °C for 1 h with type I collagenase (Sigma, USA), and hair follicles were stripped and placed in DMEM/F-12 (Gibco, USA) supplemented with 10% foetal bovine serum (FBS, Corning, USA). The cells were grown until the third generation for subsequent experiments.

Mouse MODE-K cells (iCell, China) were cultured in DMEM (Gibco, USA) supplemented with 10% FBS. The inflammation model was induced by incubation with LPS (Sigma, USA) for 24 h. The cells were incubated with Exos, Hy-Exos, Exos + 740 Y-P (25 μg/mL, MedChemExpress, USA), or Hy-Exos + 740 Y-P for 24 h for subsequent experiments.

### Adipogenic and osteogenic differentiation

When third-generation HF-MSCs were in the logarithmic phase of growth, the medium was replaced to induce adipogenic or osteogenic differentiation. Oil red O (Sigma, USA) and alizarin red (Sigma, USA) were used to stain HF-MSCs to observe osteogenesis and adipogenicity.

### Flow cytometry analysis

HF-MSCs were incubated with antibodies against CD105 (0.2 mg/mL, 120407, BioLegend, USA), CD90 (0.5 mg/mL, 140303, BioLegend, USA), CD45 (0.5 mg/mL, 103107, BioLegend, USA), CD31 (0.5 mg/mL, 160211, BioLegend, USA), and CD29 (0.5 mg/mL, 102205, BioLegend, USA) or the corresponding isotype control antibodies for 25 min. Flow cytometry was used to analyse the HF-MSCs.

### Extraction and identification of Exos and Hy-Exos

When the third-generation HF-MSCs reached 80% confluence, exosome-free medium was used. The medium consisted of DMEM/F-12 and 10% exosome-free FBS (Gibco, USA). After incubating the cells (21% oxygen) for 24 h, the supernatants were collected. HF-MSCs subjected to the same treatments were incubated under anoxic conditions (1% oxygen) for 24 h, after which the supernatants were collected. The supernatants were centrifuged at 300 × g for 10 min, 2000 × g for 10 min and 10000 × g for 30 min. Ultracentrifugation was performed twice at 100000 × g for 70 min, and the precipitates were resuspended in PBS. The morphology of the Exos and Hy-Exos was observed by TEM (Hitachi, Japan), and ZetaView (Particle Metrix, Germany) was used to measure and analyse the diameters. CD63, TSG101, HSP70 and Calnexin expression were detected by western blotting.

### Evaluation of Exos and Hy-Exos uptake

PKH26 (Sigma, USA)-labelled Exos and Hy-Exos were cocultured with MODE-K cells for 24 h. PKH67 (Sigma, USA) was used to stain the MODE-K cells. After fixation with 4% paraformaldehyde and staining with DAPI (Beyotime, China), the MODE-K cells were observed with a laser confocal microscope (Zeiss, Germany).

### Cell proliferation and cell viability assessments

Cell proliferation was analysed with an EdU assay kit (Beyotime, China). A mixture of equal volumes of culture medium and EdU working solution was incubated with MODE-K cells at 37 °C for 2 h. Then, 4% paraformaldehyde fixation was performed, followed by sequential washing and permeabilization. The click reaction solution was added to each sample and incubated for 30 min, and then Hoechst staining solution (Beyotime, China) was added. A fluorescence microscope (Nikon, Japan) was used to observe the MODE-K cells. Cell viability was determined by the CCK-8 assay (MedChemExpress, USA). Briefly, MODE-K cells were incubated with CCK-8 reagent at 37 °C for 2 h, and the absorbance of the MODE-K cells in each group was measured at 450 nm.

### Measurement of the mitochondrial membrane potential

A JC-1 staining kit (Beyotime, China) was used to determine the mitochondrial membrane potential. Briefly, a mixture of equal volumes of cell culture medium and JC-1 staining working solution was incubated with MODE-K cells at 37 °C for 20 min. MODE-K cells were then observed by a laser confocal microscope. ImageJ (National Institutes of Health, USA) was applied to calculate the ratio of aggregates (red) to monomers (green).

### Measurement of mitochondrial ROS production

The accumulation of mitochondrial ROS was analysed using MitoSOX Red fluorescent dye (Invitrogen, USA). Briefly, the MitoSOX working solution was added to MODE-K cells at 37 °C for 30 min. A laser confocal microscope was used to observe the cells, and ImageJ was used to analyse the fluorescence intensity.

### Measurement of ATP contents

An ATP assay kit (Beyotime, China) was used to determine ATP concentrations in MODE-K cells. Briefly, the proper amount of lysis buffer was used to lyse the cells, and the supernatants were centrifuged at 12000 × g for 5 min at 4 °C for subsequent analysis. The standard solution or MODE-K cells lysate was added to the ATP assay working solution, and the ATP levels were obtained from the standard curve after determining the RLU values.

### mRFP-EGFP-LC3 adenovirus transduction

Diluted adenovirus culture medium (1/2 volume) was added to MODE-K cells at a 0, 20, 40, 60, 80, or 100 multiplicity of infection (MOI). The cells were infected at 37 ºC for 4 h, after which the medium was replenished to a normal volume. After 24 h of infection, the infection efficiency was observed to determine the optimal MOI. LPS was added to the MODE-K cells to induce inflammation. The cells were incubated with Exos, Hy-Exos, Exos + 740 Y-P or Hy-Exos + 740 Y-P for 24 h, and the cells were observed via laser confocal microscopy.

### Colocalization of mitochondria and lysosomes

MODE-K cells were incubated with a mitochondrial green fluorescent probe (Beyotime, China) (1:20000) at 37 °C for 30 min and a lysosomal red fluorescent probe (Beyotime, China) (1:15000) at 37 °C for 25 min. Hoechst staining solution was added at 37 °C for 25 min, and the cells were observed with a laser confocal microscope.

### DEMs identification and enrichment analysis

Total RNA was extracted from Exos and Hy-Exos using an exoRNeasy Midi Kit (Qiagen, Germany). An Agilent 2100 Bioanalyzer (Agilent Technologies, USA) was used to evaluate the RNA quality. After the quality test, the TruSeq Small RNA Sample Prep Kit (Illumina, USA) was utilized to construct the library. The insert size of the library was analysed with an Agilent 2100 Bioanalyzer. Sequencing was performed on a NovaSeq 6000 System (Illumina, USA) at Shanghai Biotechnology Corporation. DEMs were identified using the edgeR package. *P* < 0.05 and |log2-fold change| >1 were selected as thresholds for each group.

### Cell transfection

The HF-MSCs were transfected with miR-214-3p mimics and NC mimics (Shanghai GenePharma Co., Ltd., China), and the Hy-HF-MSCs were transfected with miR-214-3p inhibitors and NC inhibitors (Shanghai GenePharma Co., Ltd., China) using LipofectamineTM 3000 Transfection Reagent (Invitrogen, USA). Exos and Hy-Exos were extracted and added to LPS-treated MODE-K cells for subsequent experiments.

### Experimental mouse UC model establishment and treatment

To establish the UC model, the mice were given 2.0% DSS (MP, Canada) for 7 days. The mice were randomly divided into 4 groups: the control, UC, Exos and Hy-Exos groups. Saline (control group and UC group), PKH26-stained Exos (Exos group, 100 μg), and PKH26-stained Hy-Exos (Hy-Exos group, 100 μg) were injected via the tail vein on days 8 and 10. Colon tissues were collected on day 14 for subsequent experiments. The determination of DAI scores in mice involved monitoring changes in body weight, faecal traits, and the degree of haematochezia.

### HE staining

After fixation with 4% paraformaldehyde, the colon tissues were dehydrated, embedded, cut into 5-μm-thick slices, and stained with haematoxylin-eosin. The sections were observed with a microscope (Olympus, Japan).

### Immunofluorescence staining

After fixation with 4% paraformaldehyde, the HF-MSCs and MODE-K cells were permeabilized and blocked. The cells were incubated with anti-CD34 (1:200, AF5149, Affinity), anti-CK15 (1:50, 60247-1-Ig, Proteintech), anti-LC3B (1:100, CY5992, Abways) and anti-COX IV (1:200, 66110-1-Ig, Proteintech) antibodies at 4 °C. After the colon tissues were fixed, the tissues were embedded to prepare frozen sections. The sections were processed with anti-Claudin1 (1:50, ab307692, Abcam), anti-Occludin (1:1000, 66378-1-Ig, Proteintech), anti-Beclin1 (1:200, 66665-1-Ig, Proteintech), anti-p62 (1:200, AF5384, Affinity), anti-LC3B (1:100, CY5992, Abways) and anti-COX IV (1:200, 66110-1-Ig, Proteintech) antibodies at 4 °C. The next day, the corresponding secondary antibodies were added to the cells and sections, the sections were stained with DAPI, and laser confocal microscopy was used to visualize the sections.

### Quantitative real-time PCR

Total RNA was extracted from MODE-K cells and colon tissues with TRIzol (Invitrogen, USA), an exoRNeasy Midi Kit was used to extract total RNA from Exos and Hy-Exos, and the concentration was determined. A reverse transcription kit (Roche, Switzerland) was used to reverse transcribe total RNA into cDNA, and a SYBR Green kit (Roche, Switzerland) was used to perform quantitative RT-PCR. After normalization to β-actin gene expression, relative gene expression was analysed by the 2^-ΔΔCT^ method. The RT-PCR primers used were showed in Table S1.

### Western blotting analysis

RIPA lysis buffer (with protease and phosphatase inhibitors) was used to lyse HF-MSCs, Exos, Hy-Exos, MODE-K cells and frozen colon tissues. The processed protein samples were electrophoresed on 7.5%, 10%, 12.5% and 15% SDS-PAGE gels, and the proteins in the gels were transferred to polyvinylidene fluoride membranes (Millipore, USA). Then, the membranes were blocked and incubated with a variety of primary antibodies at 4 °C. The primary antibodies used were against SOX9 (1:1000, CY5400, Abways), CK19 (1:5000, ab133496, Abcam), CD63 (1:1000, ab217345, Abcam), TSG101 (1:5000, ab125011, Abcam), HSP70 (1:1000, ab181606, Abcam), Calnexin (1:10000, 66903-1-Ig, Proteintech), PI3K (1:1000, ab191606, Abcam), p-PI3K (1:500, ab182651, Abcam), AKT (1:10000, ab179463, Abcam), p-AKT (1:1000, ab192623, Abcam), mTOR (1:10000, ab134903, Abcam), p-mTOR (1:5000, ab109268, Abcam), Claudin1 (1:1000, ab307692, Abcam), Occludin (1:1000, ab216327, Abcam), Drp1 (1:1000, ab184247, Abcam), Fis1 (1:2000, ab229969, Abcam), Mfn1 (1:1000, ab221661, Abcam), Mfn2 (1:5000, ab124773, Abcam), OPA1 (1:1000, YN2976, Immunoway), Beclin1 (1:2000, ab207612, Abcam), p62 (1: 10000, ab109012, Abcam), LC3B (1:1000, CY5992, Abways), HSP60 (1:1000, ab190828, Abcam), TOMM20 (1:5000, ab186735, Abcam), GAPDH (1:5000, T0004, Affinity), β-actin (1:5000, AB0061, Abways). The next day, the membranes were incubated with the corresponding secondary antibodies, and ECL (Beyotime, China) was used to detect the bands, which were subsequently analysed using ImageJ.

### *Ex vivo* organ distribution of Exos and Hy-Exos in mice

To track the distribution of Exos and Hy-Exos in mice, DiR dye (Invitrogen, USA) was used for staining. Briefly, Exos and Hy-Exos were incubated with DiR (10 mM) for 30 min and then extracted by ultracentrifugation as previously described. The mice were injected with Exos and Hy-Exos via the tail vein. After 12 h, the distribution of Exos and Hy-Exos was observed using an *in vivo* imaging system (Night OWL II LB983, Germany). Fluorescence images of the heart, liver, spleen, lung, kidney, intestine and colon were acquired and analysed separately.

### Biochemical analysis

Colon tissues were washed in precooled saline, homogenized, and centrifuged, and the supernatants were collected. MPO (Nanjing Jiancheng, China), CAT (Nanjing Jiancheng, China), GSH (Nanjing Jiancheng, China) and MDA (Nanjing Jiancheng, China) assays were performed.

### TEM

Fresh colon tissues (1 mm^3^) were fixed in 2.5% glutaraldehyde and 1% osmium tetroxide. The tissues were dehydrated in gradient ethanol solutions, embedded and sectioned. The sections were stained with uranyl acetate and lead citrate. TEM was used for visualization.

### Statistical analyses

All of the data were expressed as the mean ± SD. SPSS 25.0 and GraphPad 9.0 were used for statistical analysis and presentation. One-way ANOVA with the Bonferroni multiple comparison test was used for statistical analysis. *P* < 0.05 indicated that a difference was statistically significant.

## Supplementary Material

Supplementary figures and table.

## Author contributions

Shizhu Jin and Ning Li conceived and designed the experiments. Ning Li, Lei Zhao, Xinyu Geng, Jingyang Liu, and Xu Zhang performed the experiments. Ying Hu, Jihan Qi, Hongliang Chen, Jiawei Qiu, and Xiaoyu Zhang analysed the experimental data. Ning Li wrote the paper.
