# Supplementary material for: Stimulation by exosomes from hypoxia-preconditioned hair follicle mesenchymal stem cells facilitates mitophagy by inhibiting the PI3K/AKT/mTOR signaling pathway to alleviate ulcerative colitis
Source: Theranostics. 2024 Jul 8;14(11):4278–96. doi: 10.7150/thno.96038 (PMC11303078; doi:10.7150/thno.96038)
Supplement: Supplementary file 1 — Supplementary figures and table. [file thnov14p4278s1.pdf]

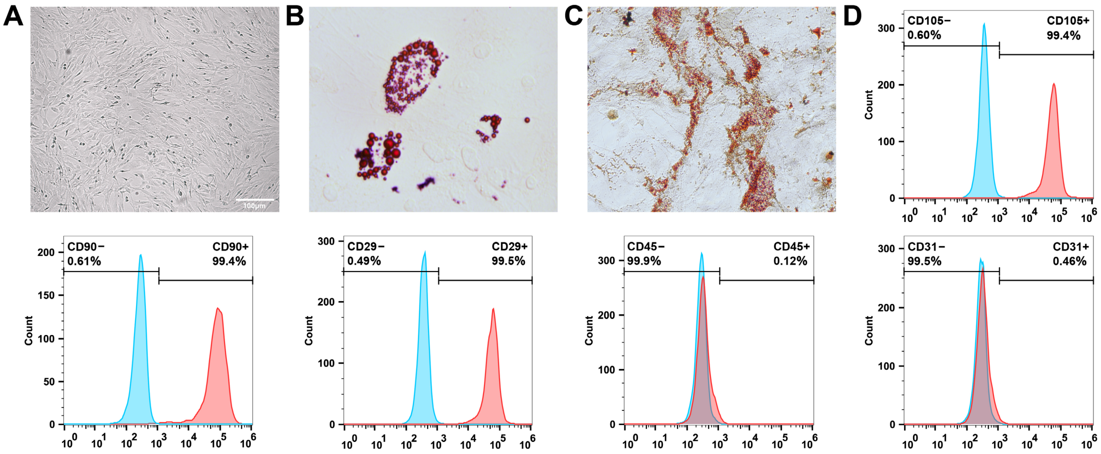

Figure S1. Characterization of Hy-HF-MSCs. A. Cell morphology of Hy-HF-MSCs. Scale bar = 100 μm. B. Adipogenic differentiation of Hy-HF-MSCs. C. Osteogenic differentiation of Hy-HF-MSCs. D. Flow cytometry was used to detect Hy-HF-MSC-specific antigenic markers.

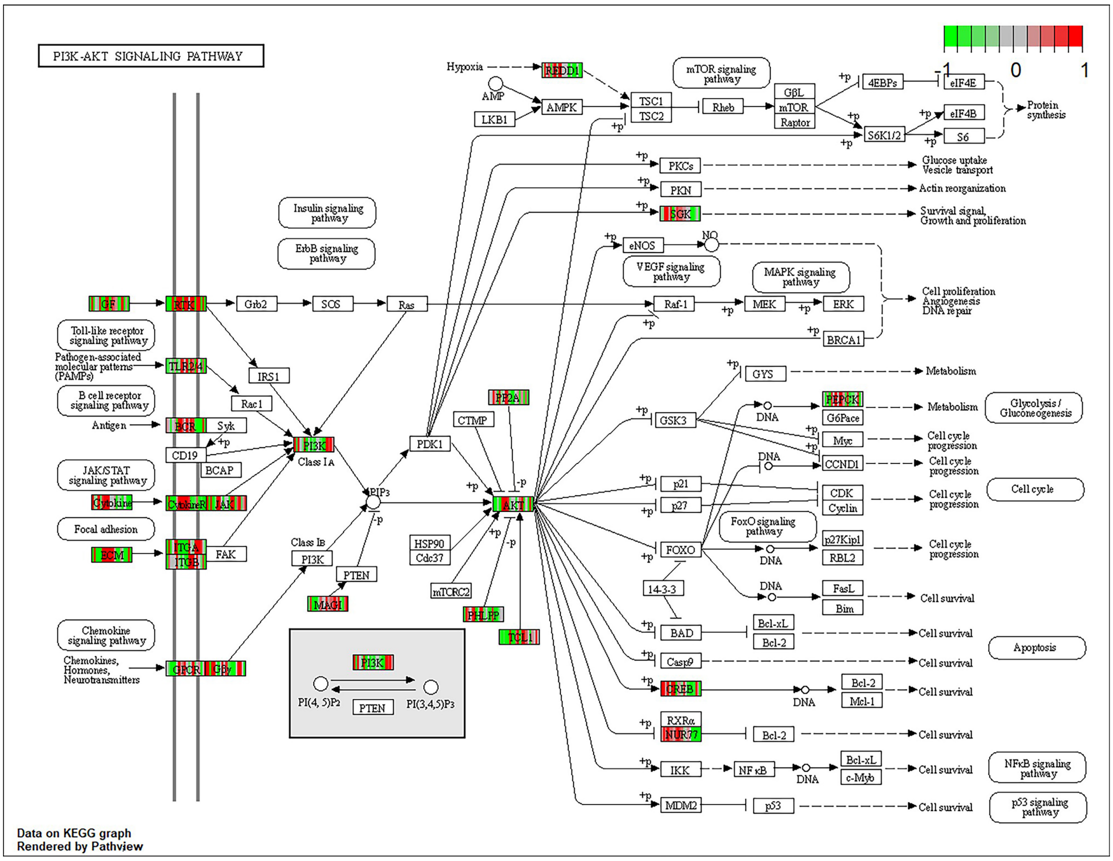

Figure S2. Enrichment results of DEGs in the PI3K-AKT signaling pathway.

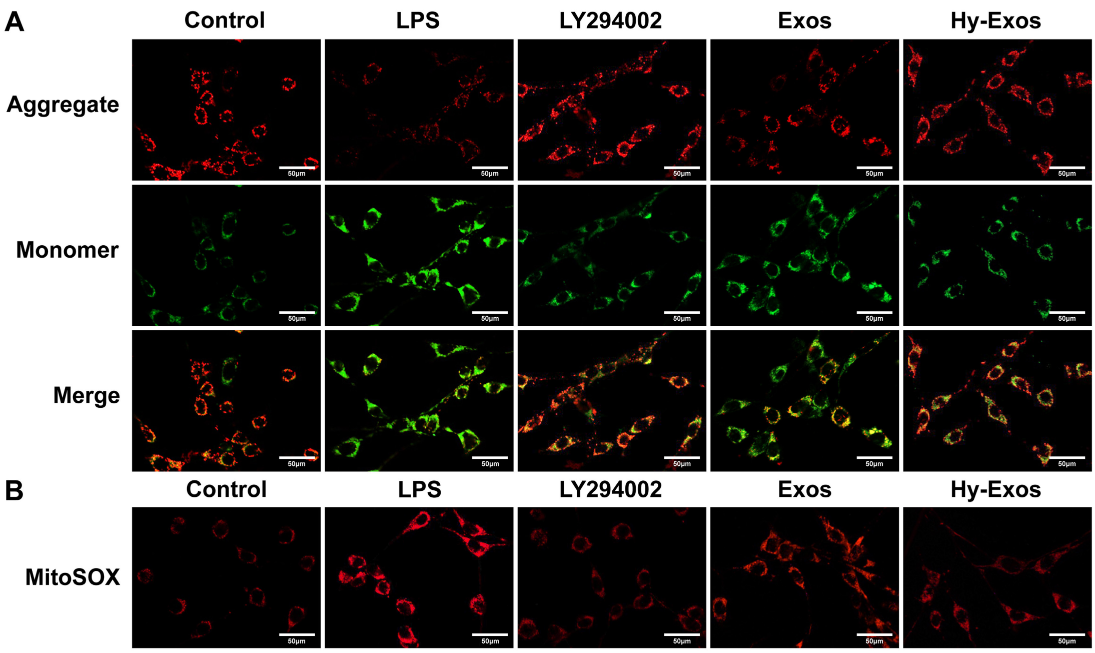

Figure S3. The PI3K inhibitor LY294002 alleviated mitochondrial dysfunction in MODE-K cells. A. Mitochondrial membrane potential in MODE-K cells as measured by a JC-1 staining kit. Scale bar = 50 μm. B. Mitochondrial ROS was detected in MODE-K cells. Scale bar = 50 μm.

Table S1. Primer sequences for quantitative real-time PCR.

Genes	Forward primer (5'-3')	Reverse primer (5'-3')
IL-1β	GCAACTGTTCCTGAACTCAACT	ATCTTTTGGGGTCCGTCAACT
TNF-α	CCTCTCTCTAATCAGCCCTCTG	GAGGACCTGGGAGTAGATGAG
IL-4	GCCATATCCACGGATGCGACAA	GGTGTTCTTCGTTGCTGTGAGGA
IL-10	GCTCTTACTGACTGGCATGAG	CGCAGCTCTAGGAGCATGTG
β-actin	GGCTGTATTCCCCTCCATCG	CCAGTTGGTAACAATGCCATGT
miR-92b-3p	GTCCGCTATTGCACTCGTCCCGGCCTCC	GTGCGTGTCGTGGAGTC
miR-484	GCGTCAGGCTCAGTCCCCT	AGTGCAGGGTCCGAGGTATT
miR-214-3p	ACGAGAACACAGCAGGCACAG	ATCCAGTGCAGGGTCCGAGG
miR-30a-5p	AACGAGACGACGACAGAC	TGTAAACATCCTCGACTGGAAG
miR-205-5p	CGTCCTTCATTCCACCGG	AGTGCAGGGTCCGAGGTATT
U6	CCGTATGACCTCCTTCCACAGA	TCTGTCCACCTCTGAAACCAGG
